# Association between surgeon experience, level of supervision, and outcomes after hip fracture

**DOI:** 10.1302/2633-1462.76.BJO-2025-0321.R1

**Published:** 2026-06-04

**Authors:** En Lin Goh, May Ee Png, David Metcalfe, Juul Achten, Duncan Appelbe, Xavier L. Griffin, Jonathan A. Cook, Matthew L. Costa, WHiTE Investigators

**Affiliations:** 1 Oxford Trauma and Emergency Care, Nuffield Department of Orthopaedics, Rheumatology and Musculoskeletal Sciences, University of Oxford, Oxford, UK; 2 Nuffield Department of Primary Care Health Sciences, University of Oxford, Oxford, UK; 3 Bone and Joint Health, Blizard Institute, Queen Mary University of London, London, UK; 4 Oxford Clinical Trials Research Unit, Nuffield Department of Orthopaedics, Rheumatology and Musculoskeletal Sciences, University of Oxford, Oxford, UK

**Keywords:** Hip fracture, Training, Residents, Consultants, hip fractures, Operating surgeon, EQ-5D-5L, revision surgery, blood transfusions, Cox proportional hazards regression, respiratory tract infection, prospective cohort study, cerebrovascular accident, myocardial infarction

## Abstract

**Aims:**

Patients with a hip fracture are treated by surgeons at various stages of training, under different levels of supervision. However, it is unclear whether their experience and the level of supervision are associated with patient outcomes. The aim of this study was to investigate the relationship between operating surgeon experience and level of supervision, and the patients’ subsequent quality of life (QoL), mortality, and complications after surgery for a hip fracture.

**Methods:**

A multicentre, prospective cohort study of patients aged 60 years and older with a hip fracture in the UK. Primary exposure was operating surgeon grade and level of supervision. Outcomes were health-related quality of life (HRQoL; EuroQol five-dimension five-level questionnaire (EQ-5D-5L)), mortality, and complications at four months. Linear and Cox proportional hazards regression models were fitted to assess the relationship between operating surgeon grade and level of supervision, and HRQoL, mortality, and complications.

**Results:**

Among 24,523 patients with a hip fracture, there were 12,702 consultant-performed and 11,365 resident-performed operations. Operations performed by supervised residents had better recovery of EQ-5D-5L (mean difference (MD) 0.02, 95% CI 0.01 to 0.03; p < 0.001), a similar risk of mortality (HR 0.92, 95% CI 0.84 to 1.01; p = 0.083), but higher risks of reoperation (HR 1.29, 95% CI 1.06 to 1.57; p = 0.009), blood transfusion (HR 1.44, 95% CI 1.28 to 1.62; p < 0.001), acute kidney injury (HR 1.72, 95% CI 1.46 to 2.04; p < 0.001), lower respiratory tract infection (HR 1.22, 95% CI 1.10 to 1.35; p < 0.001), cerebrovascular accident (HR 1.98, 95% CI 1.33 to 2.95, p < 0.001), and myocardial infarction (HR 1.91, 95% CI 1.33 to 2.74; p < 0.001), compared with operations performed by consultants. Operations performed by unsupervised residents had better recovery of EQ-5D-5L (MD 0.02, 95% CI 0.00 to 0.03; p = 0.008), a similar risk of mortality (HR 0.92, 95% CI 0.81 to 1.06; p = 0.260), but a higher risk of blood transfusion (HR 1.54, 95% CI 1.33 to 1.80; p < 0.001) compared with operations performed by consultants.

**Conclusion:**

Operations performed by residents were associated with a better recovery of QoL, similar mortality, but slightly more complications compared with consultants. This highlights the need for tailored supervision and structured training to optimize outcomes and ensure patient safety.

Cite this article: *Bone Jt Open* 2026;7(6):744–752.

## Introduction

A hip fracture is the most common serious injury in older adults.^1,2^ An estimated 80,000 hip fractures occur every year in the UK,^3,4^ with projections indicating a rise in the current annual incidence to over 100,000 hip fractures per year in the next decade.^5^ Within the UK healthcare system, almost everyone with a hip fracture will undergo surgery.^3^ This involves arthroplasty of the hip joint with a hip hemiarthroplasty or total hip arthroplasty, or fixation of the fracture with a sliding hip screw, cephalomedullary nail, or cannulated screws.^6^

These operations are performed by surgeons at various stages in their training, under different levels of supervision. There is evidence to suggest that patients treated by resident surgeons have a higher risk of death and further surgery, but this is contentious.^7-13^ A recent study of 1.3 million operations observed comparable mortality and morbidity between residents and consultants, although orthopaedic procedures accounted for a small proportion of the cases.^14^ Most of these studies were retrospective in design, conducted at a single institution, and/or had a small population size, thus limiting their generalizability.^7,8,11-13,15^ Furthermore, none have considered quality of life (QoL), which is the most important outcome to patients after a hip fracture.^16^

It is evident that there must be a balance between protecting training opportunities to develop surgeons who are competent, and limiting the exposure of patients to potential risks during the training process to minimize harm.^17^ The aim of this study was to investigate the relationship between operating surgeon grade and level of supervision, and QoL, mortality, and complications in patients with a hip fracture. We hypothesized that operations performed by residents under supervision would have equivalent outcomes to those performed by consultants. To achieve this, we set out to compare outcomes between operations performed by 1) consultants and residents; and 2) residents under different levels of supervision.

## Methods

### Study design and setting

The World Hip Trauma Evaluation (WHiTE) study was a multicentre, prospective cohort study across 77 participating NHS hospitals in England, Wales, and Northern Ireland.^18^ Patients were followed up for four months after surgery with telephone interviews and postal questionnaires. Recruitment for the study commenced on 8 May 2014, and finished on 29 July 2021, with follow-up to 26 November 2021.

### Participants

Patients were eligible for inclusion if they were aged 60 years or older and received operative treatment for their hip fracture. On enrolment, patients received treatment under a standardized care pathway based on the National Institute for Health and Care Excellence Hip Fracture Guidelines (CG124).^6^ The demographic details of the cohort are described in Table I.

**Table I.** Baseline characteristics of patients who had their operations performed by consultants compared with residents and supervised residents compared with unsupervised residents.

Characteristic	Consultant-performed vs resident-performed operations		Resident-performed – supervised vs resident-performed – unsupervised operations	
	Consultant-performed (n = 12,702)	Resident-performed (n = 11,365)	Resident-performed – supervised (n = 8,309)	Resident-performed – unsupervised (n = 3,056)
**Mean age, yrs (SD)**	82.5 (8.6)	83.3 (8.2)	83.4 (8.2)	83.1 (8.2)
**Sex, n (%)**				
Male	3,840 (30.2)	3,351 (29.5)	2,494 (30.0)	857 (28.0)
Female	8,862 (69.8)	8,014 (70.5)	5,815 (70.0)	2,199 (72.0)
Missing	0 (0.0)	0 (0.0)	0 (0.0)	0 (0.0)
**Regular smoker, n (%)**				
Yes	1,108 (8.7)	1,004 (8.8)	735 (8.8)	269 (8.8)
No	10,868 (85.6)	9,865 (86.8)	7,341 (88.3)	2,524 (82.6)
Missing	726 (5.7)	496 (4.4)	233 (2.8)	263 (8.6)
**Weekly alcohol consumption, n (%)**				
0 to 7 units	10,575 (83.3)	9,662 (85.0)	7,171 (86.3)	2,491 (81.5)
8 to 14 units	765 (6.0)	671 (5.9)	507 (6.1)	164 (5.4)
15 to 21 units	296 (2.3)	207 (1.8)	152 (1.8)	55 (1.8)
> 21 units	305 (2.4)	292 (2.6)	220 (2.6)	72 (2.4)
Missing	761 (6.0)	533 (4.7)	259 (3.1)	274 (9.0)
**Diabetic, n (%)**				
Yes	1,845 (14.5)	1,725 (15.2)	1,276 (15.4)	449 (14.7)
No	10,150 (79.9)	9,195 (80.9)	6,839 (82.3)	2,356 (77.1)
Missing	707 (5.6)	445 (3.9)	194 (2.3)	251 (8.2)
**Renal failure, n (%)**				
Yes	822 (6.5)	962 (8.5)	776 (9.3)	186 (6.1)
No	11,158 (87.8)	9,947 (87.5)	7,331 (88.2)	2,616 (85.6)
Missing	722 (5.7)	456 (4.0)	202 (2.4)	254 (8.3)
**Cognitive impairment, n (%)**				
Yes	3,896 (30.7)	3,785 (33.3)	2,858 (34.4)	927 (30.3)
No	8,238 (64.9)	7,143 (62.9)	5,109 (61.5)	2,034 (66.6)
Missing	568 (4.5)	437 (3.8)	342 (4.1)	95 (3.1)
**ASA grade, n (%)** ^19^				
I	284 (2.2)	164 (1.4)	104 (1.3)	60 (2.0)
II	2,969 (23.4)	2,495 (22.0)	1,673 (20.1)	822 (26.9)
III	6,951 (54.7)	6,485 (57.1)	4,827 (58.1)	1,658 (54.3)
IV	1,868 (14.7)	1,648 (14.5)	1,310 (15.8)	338 (11.1)
V	40 (0.3)	30 (0.3)	27 (0.3)	3 (0.1)
Missing	590 (4.6)	543 (4.8)	368 (4.4)	175 (5.7)
**Fracture type, n (%)**				
Femoral neck – undisplaced (B1)	936 (7.4)	689 (6.1)	428 (5.2)	261 (8.5)
Femoral neck – displaced (B3)	8,017 (63.1)	6,948 (61.1)	5,464 (65.8)	1,484 (48.6)
Trochanteric – simple (A1)	1,030 (8.1)	1,338 (11.8)	882 (10.6)	456 (14.9)
Trochanteric – unstable (A2)	1,789 (14.1)	1,583 (13.9)	1,004 (12.1)	579 (18.9)
Trochanteric – transtrochanteric (A3)	401 (3.2)	408 (3.6)	276 (3.3)	132 (4.3)
Subtrochanteric	524 (4.1)	395 (3.5)	252 (3.0)	143 (4.7)
Missing	5 (0.0)	4 (0.0)	3 (0.0)	1 (0.0)
**Operation type, n (%)**				
Sliding hip screw	2,618 (20.6)	2,852 (25.1)	1,743 (21.0)	1,109 (36.3)
Cephalomedullary nail	1,517 (11.9)	1,111 (9.8)	796 (9.6)	315 (10.3)
Cannulated screws	1,635 (12.9)	424 (3.7)	287 (3.5)	137 (4.5)
Hip hemiarthroplasty	5,714 (45.0)	5,479 (48.2)	4,120 (49.6)	1,359 (44.5)
Total hip arthroplasty	291 (2.3)	339 (3.0)	208 (2.5)	131 (4.3)
Missing	927 (7.3)	1,160 (10.2)	1,155 (13.9)	5 (0.2)
**Mean pre-injury EQ-5D-5L (SD)**	0.76 (0.24)	0.73 (0.25)	0.73 (0.25)	0.72 (0.26)

ASA, American Society of Anesthesiologists; EQ-5D-5L, EuroQol five-dimension five-level questionnaire.

### Ethics approval

Ethics approval was granted by the London-Camberwell St Giles Research Ethics Committee.^18^ This study was registered with the National Institute of Health Research Portfolio (UKCRN ID12351) and the ISRCTN registry (ISRCTN63982700). Written consent to participate in the study was obtained from all patients. Patients who lacked capacity to consent to participate were still included in consultation with their carers.

### Data sources

The WHiTE dataset contains data on a core outcome set of patient-reported outcome measures (PROMs) in addition to variables that are routinely measured by the UK National Hip Fracture Database.^3^ The full list of variables and outcomes collected as part of the study has been described previously.^18^ Data were stored on the OpenClinica V3.7 data collection system (OpenClinica LLC, USA).

### Variables

The primary variables of interest were operating surgeon grade and level of supervision. Operating surgeon grade was classified as consultant-performed (consultant doing the operation) or resident-performed (resident doing the majority or all of the operation with or without the consultant present in the operating theatre). Level of supervision for resident-performed operations was classified as supervised (consultant present in the operating theatre and either scrubbed or unscrubbed) or unsupervised (consultant not present in the operating theatre). The grades and definitions of surgeons in the consultant and resident groups are described in Supplementary Table i. The covariates selected for inclusion in the statistical models were specified a priori and are described in Supplementary Table ii. The covariates considered included the patient’s demographic details, pre-injury health-related QoL (HRQoL), comorbidities, fracture type, and operation type, which have previously been shown to influence the recovery of QoL, mortality, and complications after hip fracture.^20-24^

### Outcomes

The primary outcome of interest was HRQol, as measured with the EuroQol five-dimension five-level questionnaire (EQ-5D-5L) at four months after surgery. EQ-5D-5L is a validated self-administered PROM based on a five-dimension health status descriptive system and a separate visual analogue scale (EQ-VAS) for overall health state (0 to 100 scale).^25^ The responses from the descriptive system were converted into an overall score using a published utility algorithm for the UK population.^26,27^ The minimal clinically important difference in EQ-5D-5L is considered to be 0.05.^28^ The secondary outcomes of interest were all-cause mortality and complications during the four months after surgery. The complications evaluated were prespecified and are listed in Supplementary Table iii.

### Statistical analysis

The baseline characteristics of the study cohort were reported as means and SDs for continuous measures, and proportions (binary) as appropriate. HRQoL, mortality, and complications associated with 1) consultant-performed and resident-performed operations; 2) resident-performed – supervised and resident performed – unsupervised operations; and 3) consultant-performed, resident-performed – supervised, and resident-performed unsupervised operations were compared. Linear regression models were used to examine HRQoL (EQ-5D-5L), and Cox proportional hazards regression models were used to examine mortality and complications. The continuous variable, age was modelled with restricted cubic splines to allow for non-linear effects. The assumption of proportional hazards was assessed with Schoenfeld residual tests for each covariate and the overall model using the method described by Grambsch and Therneau.^29^ Where the proportional hazards assumption was violated, 1) stratification of the covariate was performed to fit a separate baseline hazard function for each level in the stratification covariate; and 2) a time interaction was incorporated into the model for the variable. The change in the average hazard ratios (HRs) over time for time-dependent variables are reported in Supplementary Figures a to d. Death-adjusted EQ-5D-5L was used; patients who died before completing the post-injury EQ-5D-5L were assigned a value of zero.^30^ Analyses were conducted on the complete case dataset. The results are presented as mean differences for the linear regression models and HRs for the Cox proportional hazards regression models, with corresponding 95% CIs and p-values. The significance threshold was two-sided p < 0.05. Statistical analyses were performed with R statistical software (v4.5.0; R Core Team 2025, R Foundation for Statistical Computing, Austria). EQ-5D-5L utility score was mapped from the five dimensions using the ‘eq5d’ R package.^31^ Linear regression models were fitted using the ‘lme4’ R package.^32^ Cox proportional hazards regression models were fitted using the ‘survival’ R package.^33^

## Results

A total of 24,523 patients were enrolled, for whom data on the operating surgeon grade and level of supervision were available in 24,067 (98.1%) patients and complete follow-up in 22,228 (90.6%) patients. Of these, 12,702 (52.8%) patients had their operation performed by a consultant and 11,365 patients (47.2%) by a resident.

### Health-related quality of life

Resident-performed operations were associated with a small but significantly better recovery of EQ-5D-5L compared with consultant-performed operations (mean difference 0.02, 95% CI 0.01 to 0.03; p < 0.001). Resident-performed – unsupervised operations were associated with similar recovery of EQ-5D-5L compared with resident-performed – supervised operations (mean difference –0.01, 95% CI –0.02 to 0.01; p = 0.393). Compared with consultant-performed operations, recovery of EQ-5D-5L was better with resident-performed – supervised operations (mean difference 0.02, 95% CI 0.01 to 0.03; p < 0.001) and resident-performed – unsupervised operations (mean difference 0.02, 95% CI 0.00 to 0.03; p = 0.008).

### Mortality

The adjusted HRs for all-cause mortality for patients who had their operation performed by consultants compared with residents and supervised compared with unsupervised residents are reported in Table II. Resident-performed operations had a similar risk of all-cause mortality compared with consultant-performed operations (HR 0.92, 95% CI 0.85 to 1.00; p = 0.063). Resident-performed – unsupervised operations had a similar risk of all-cause mortality compared with resident-performed – supervised operations (HR 1.01, 95% CI 0.88 to 1.16; p = 0.930). Compared with consultant-performed operations, the risk of mortality was similar for resident-performed – supervised operations (HR 0.92, 95% CI 0.84 to 1.01; p = 0.083) and resident-performed – unsupervised operations (HR 0.92, 95% CI 0.81 to 1.06; p = 0.260).

**Table II.** Cumulative incidences of mortality and complications and adjusted hazard ratios for operations performed by consultants compared with residents and supervised residents compared with unsupervised residents.

Outcome	Cumulative incidence, % (95% CI)		HR	p-value	Cumulative incidence, % (95% CI)		HR	p-value
	Consultant-performed (n = 12,702)	Resident-performed (n = 11,365)			Resident-performed – supervised (n = 8,309)	Resident-performed – unsupervised (n = 3,056)		
Mortality (all-cause)	12.5 (11.9 to 13.1)	12.5 (11.9 to 13.1)	0.92 (0.85 to 1.00)	0.063	12.9 (12.1 to 13.6)	11.5 (10.3 to 12.6)	1.01 (0.88 to 1.16)	0.930
Reoperation (all-cause)	2.6 (2.3 to 2.9)	2.9 (2.6 to 3.2)	1.15 (0.96 to 1.38)	0.130	3.3 (2.9 to 3.7)	1.7 (1.2 to 2.1)	0.61 (0.44 to 0.84)	0.003
Prosthesis dislocation	1.6 (1.4 to 1.9)	1.7 (1.4 to 2.0)	1.13 (0.83 to 1.53)	0.450	1.8 (1.5 to 2.2)	1.2 (0.6 to 1.7)	0.85 (0.50 to 1.45)	0.550
Fixation failure	0.9 (0.6 to 1.2)	1.0 (0.7 to 1.3)	1.03 (0.64 to 1.65)	0.910	1.1 (0.7 to 1.4)	0.9 (0.5 to 1.4)	0.70 (0.33 to 1.46)	0.340
Periprosthetic or peri-implant fracture	0.3 (0.2 to 0.4)	0.4 (0.2 to 0.5)	1.24 (0.76 to 2.00)	0.390	0.4 (0.3 to 0.6)	0.1 (0.0 to 0.3)	0.36 (0.13 to 1.01)	0.053
Surgical site infection	3.4 (3.1 to 3.7)	3.4 (3.1 to 3.8)	0.99 (0.85 to 1.17)	0.950	3.7 (3.2 to 4.1)	2.8 (2.2 to 3.4)	0.85 (0.65 to 1.11)	0.220
Blood transfusion	5.9 (5.5 to 6.3)	8.3 (7.8 to 8.7)	1.47 (1.32 to 1.64)	< 0.001	8.0 (7.5 to 8.6)	8.9 (7.9 to 9.9)	1.07 (0.92 to 1.25)	0.380
Acute kidney injury	2.7 (2.4 to 3.0)	4.2 (3.8 to 4.5)	1.46 (1.24 to 1.72)	< 0.001	5.0 (4.6 to 5.5)	1.8 (1.3 to 2.3)	0.42 (0.30 to 0.57)	< 0.001
Lower respiratory tract infection	8.3 (7.8 to 8.7)	10.0 (9.5 to 10.6)	1.13 (1.03 to 1.25)	0.008	11.2 (10.6 to 11.9)	6.7 (5.9 to 7.6)	0.73 (0.62 to 0.86)	< 0.001
Cerebrovascular accident	0.5 (0.4 to 0.6)	0.9 (0.7 to 1.1)	1.79 (1.22 to 2.62)	0.003	1.0 (0.8 to 1.2)	0.6 (0.3 to 0.8)	0.64 (0.35 to 1.15)	0.130
Myocardial infarction	0.6 (0.4 to 0.7)	0.9 (0.8 to 1.1)	1.68 (1.19 to 2.38)	0.003	1.1 (0.9 to 1.3)	0.6 (0.3 to 0.8)	0.55 (0.31 to 0.98)	0.042
Venous thromboembolism	1.8 (1.6 to 2.1)	1.8 (1.6 to 2.0)	1.04 (0.84 to 1.29)	0.720	1.8 (1.6 to 2.1)	1.7 (1.2 to 2.1)	0.89 (0.63 to 1.25)	0.490

HR, hazard ratio.

### Complications

The adjusted HRs for complications for patients who had their operation performed by consultants compared with residents and supervised compared with unsupervised residents are reported in Table II. Compared with consultant-performed operations, resident-performed operations had higher risks of blood transfusion (HR 1.47, 95% CI 1.32 to 1.64, p < 0.001), acute kidney injury (AKI) (HR 1.46, 95% CI 1.24 to 1.72, p < 0.001), lower respiratory tract infection (LRTI) (HR 1.13, 95% CI 1.03 to 1.25, p = 0.008), cerebrovascular accident (CVA) (HR 1.79, 95% CI 1.22 to 2.62, p = 0.003), and myocardial infarction (MI) (HR 1.68, 95% CI 1.19 to 2.38, p = 0.003), but similar risks of all-cause reoperation (HR 1.15, 95% CI 0.96 to 1.38, p = 0.130), prosthesis dislocation (HR 1.13, 95% CI 0.83 to 1.53, p = 0.450), fixation failure (HR 1.03, 95% CI 0.64 to 1.65, p = 0.910), periprosthetic or peri-implant fracture (PPF) (HR 1.24, 95% CI 0.76 to 2.00, p = 0.390), surgical site infection (SSI) (HR 0.99, 95% CI 0.85 to 1.17, p = 0.950), and venous thromboembolism (VTE; HR 1.04, 95% CI 0.84 to 1.29, p = 0.720).

Compared with resident-performed – supervised operations, resident-performed – unsupervised operations had similar risks of prosthesis dislocation (HR 0.85, 95% CI 0.50 to 1.45, p = 0.550), fixation failure (HR 0.70, 95% CI 0.33 to 1.46, p = 0.340), PPF (HR 0.36, 95% CI 0.13 to 1.01, p = 0.053), SSI (HR 0.85, 95% CI 0.65 to 1.11, p = 0.220), blood transfusion (HR 1.07, 95% CI 0.92 to 1.25, p = 0.380), CVA (HR 0.64, 95% CI 0.35 to 1.15, p = 0.130), and VTE (HR 0.89, 95% CI 0.63 to 1.25, p = 0.490), but lower risks of all-cause reoperation (HR 0.61, 95% CI 0.44 to 0.84, p = 0.003), AKI (HR 0.42, 95% CI 0.30 to 0.57, p < 0.001), LRTI (HR 0.73, 95% CI 0.62 to 0.86, p < 0.001), and MI (HR 0.55, 95% CI 0.31 to 0.98, p = 0.042).

**Fig. 1:**
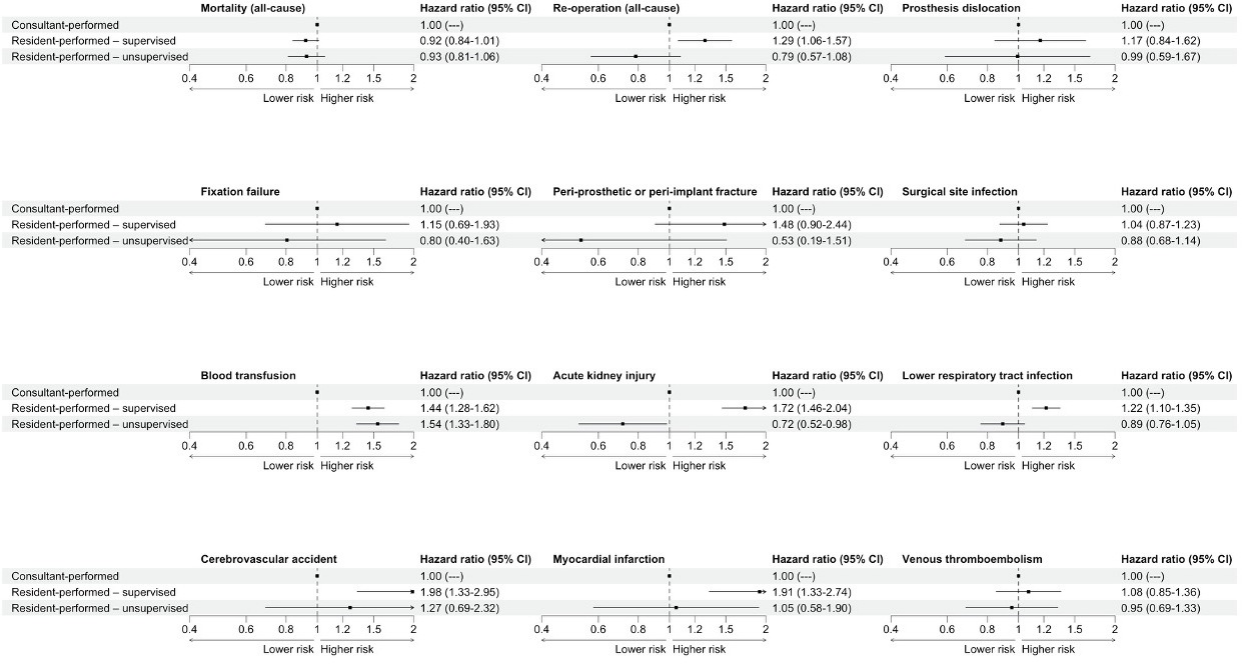
Forest plots showing the adjusted hazard ratios for all-cause mortality and complications for operations performed by consultants, supervised residents, and unsupervised residents.The reference level was set to consultant-performed (hazard ratio set to 1.00, with no corresponding 95% CI and p-value for illustrative purpose).

The adjusted HRs for all-cause mortality and complications for consultant-performed, resident-performed – supervised, and resident-performed – unsupervised operations are reported in Figure 1. Compared with consultant-performed operations, resident-performed – supervised operations had higher risks of all-cause reoperation (HR 1.29, 95% CI 1.06 to 1.57, p = 0.009), blood transfusion (HR 1.44, 95% CI 1.28 to 1.62, p < 0.001), AKI (HR 1.72, 95% CI 1.46 to 2.04, p < 0.001), LRTI (HR 1.22, 95% CI 1.10 to 1.35, p < 0.001), CVA (HR 1.98, 95% CI 1.33 to 2.95, p < 0.001), and MI (HR 1.91, 95% CI 1.33 to 2.74, p < 0.001), and similar risks of prosthesis dislocation (HR 1.17, 95% CI 0.84 to 1.62, p = 0.350), fixation failure (HR 1.15, 95% CI 0.69 to 1.93, p = 0.590), PPF (HR 1.48, 95% CI 0.90 to 2.44, p = 0.120), SSI (HR 1.04, 95% CI 0.87 to 1.23, p = 0.670), and VTE (HR 1.08, 95% CI 0.85 to 1.36, p = 0.540).

Compared with consultant-performed operations, resident-performed – unsupervised operations had a higher risk of blood transfusion (HR 1.54, 95% CI 1.33 to 1.80; p < 0.001), and similar risks of all-cause reoperation (HR 0.79, 95% CI 0.57 to 1.08; p = 0.140), prosthesis dislocation (HR 0.99, 95% CI 0.59 to 1.67; p = 0.980), fixation failure (HR 0.80, 95% CI 0.40 to 1.63; p = 0.550), PPF (HR 0.53, 95% CI 0.19 to 1.51; p = 0.230), SSI (HR 0.88, 95% CI 0.68 to 1.14; p = 0.330), LRTI (HR 0.89, 95% CI 0.76 to 1.05; p = 0.160), CVA (HR 1.27, 95% CI 0.69 to 2.32; p = 0.450), MI (HR 1.05, 95% CI 0.58 to 1.90; p = 0.870), and VTE (HR 0.95, 95% CI 0.69 to 1.33; p = 0.780), but lower risks of AKI (HR 0.72, 95% CI 0.52 to 0.98; p = 0.038).

## Discussion

In the present study, we investigated the association between surgeon grade and level of supervision, and outcome after hip fracture. QoL is the most important outcome to patients with a hip fracture, but there are no previous data on how this is influenced by the grade of the operating surgeon and level of supervision.^16,34^ This study showed that operations performed by residents were associated with better recovery of QoL compared with those performed by consultants. While statistically significant, the difference in EQ-5D-5L was small and therefore unlikely to be clinically meaningful. We also observed comparable recovery of QoL between supervised and unsupervised resident-performed operations. The differential attainment found between residents and consultants may be due to reverse causality, where the sickest patients with the least potential for recovery were operated on by consultants.^9,10^ Nevertheless, our findings indicate that residents can achieve equivalent if not superior outcomes in terms of QoL to consultants when performing hip fracture surgery.

The effect of resident involvement on mortality after hip fracture surgery has been a point of debate. There were no differences in mortality between resident- and consultant-performed operations, and between supervised and unsupervised resident-performed operations in our study. The only study to examine this relationship in the hip fracture population in the UK healthcare system was conducted by Khunda et al,^8^ who reported that patients treated by residents were 80% more likely to die compared with consultants. However, this observation has not been replicated across other healthcare systems. In their review of 1,764 patients from the American College of Surgeons National Surgical Quality Improvement Program database, Neuwirth et al^10^ found no difference in mortality following operations with and without resident involvement. A study based on the Norwegian Hip Fracture Register noted equivalent mortality between experienced and inexperienced surgeons.^9^ Prat et al^13^ observed comparable mortality between operations performed by senior residents and consultants in their single-institution series. Our data add to the current evidence base indicating that resident-performed operations do not put patients at any unnecessary risk of death.

Patients operated on by residents experienced similar risks of surgery-specific complications, but higher risks of general complications, compared with those operated on by consultants. However, residents under supervision had higher rates of both surgery-specific and general complications when compared with consultants. Specifically, they had more reoperations, blood transfusions, AKIs, LRTIs, CVAs, and MIs. These results corroborate the work of Palm et al^7^ and Authen et al,^9^ who reported more reoperations with inexperienced surgeons than experienced surgeons. It is evident that this finding is not unique to the hip fracture population. Recent data from the National Joint Registry in the UK have shown a higher risk of revision surgery following resident-performed total hip arthroplasty.^35^ The association between surgeon grade and general complications, i.e. not surgery-specific, is a novel finding that has not been documented previously.^10,36^ These data have important clinical implications given that general complications affect almost one-third of patients with a hip fracture, with a substantial negative impact on recovery of QoL.^23,37,38^

The goal of surgical training is to ensure that surgeons are competent and safe by the time they achieve independent practice as consultants. As such, it is reasonable to expect residents to have operated independently during their training. We found that unsupervised residents had fewer complications in terms of reoperations, AKIs, and LRTIs compared with supervised residents. Furthermore, unsupervised residents had fewer complications than consultants except in the case of blood transfusion. To our knowledge, this is the first study to compare operations performed by residents independently with those performed by residents with assistance and guidance from consultants, and with those performed by consultants for hip fracture. This comparison has important implications for surgical training, as it addresses the question of whether it is safe for a resident to perform hip fracture surgery independently during their training. The results of our study provide evidence that it is possible to do so with no detriment to patients.

Our work also provides insights into the modern-day system of surgical training in the UK.^39^ Concerns have been raised over a possible deficit in the quality of surgical training, which has been attributed to the decline in training opportunities and loss of autonomy experienced by residents in the operating theatre.^40,41^ These issues are not confined to the UK, having been documented across other developed healthcare systems.^42-44^ In the USA, 92% of residents undertaking the 2020 American Board of Surgery In-Training Examination reported deficits in preparation for practice despite being five months away from completing training.^44^ There appears to be a gap between the actual and expected operative performance of residents and their trainers,^43^ which correlates with a decline in confidence in their ability.^42^ Our observations refute these concerns. Unsupervised residents were able to achieve better outcomes than their supervised contemporaries and equivalent outcomes compared with their consultants, which is likely to reflect the development in their surgical skill, competence, and graduated autonomy as they progress through training.^45^ Therefore, our data support the safety and effectiveness of the current system for surgical training in the UK.

Complete follow-up was available in over 90% of patients, and the complete case population was comparable with the overall study population.^23,24,38,46^ There are, however, important limitations that should be considered. The associations identified between surgeon grade and level of supervision with QoL, mortality, and complications should not be taken as proof of a causal pathway. Although we adjusted for known confounders that have been linked to outcome after hip fracture, residual confounding may be present. There is likely reverse causality between consultant-performed and unsupervised resident-performed operations representing appropriate patient and case selection, and therefore selection bias, rather than indicating that residents performed better than consultants and without supervision. Residents would have satisfied the required competencies to perform these operations independently, and our findings should not be taken as proof that all residents can be left without supervision. The term ‘resident’ is broad and does not account for the variation in their level of experience. Additionally, the level of supervision coded may not fully reflect the spectrum of supervision that is inherent to surgical training. Comparisons with other studies is difficult because of differences in how the level of resident involvement is defined. These are inherent limitations that should be considered when interpreting any study on this topic.

In conclusion, within the UK healthcare system, patients with a hip fracture who had their operation performed by residents experienced slightly better recovery of QoL without any excess risk of mortality compared with consultants. This change in QoL was statistically significant, but unlikely to be clinically meaningful. However, these patients had higher risks of some general complications. The data in this study provide evidence to suggest that it is safe for residents to perform operations independently, with appropriate case selection. We advocate for every continued effort to encourage resident autonomy, given that this can be achieved without compromising good patient outcomes. This study provides high-quality evidence to support the safety and effectiveness of the current training system for hip fracture care in the UK. Future work evaluating longer-term outcomes after hip fracture between consultant- and resident-performed operations will be useful towards validating the present findings.

**Take home message**

- Hip fracture operations performed by resident surgeons achieve equivalent mortality rates and quality of life outcomes compared to those performed by consultant surgeons.

- While resident-performed surgeries are linked to slightly higher risks of general complications, unsupervised residents achieve excellent outcomes when paired with proper case selection.

- These findings validate the safety and effectiveness of the current UK training system, proving that fostering graduated resident autonomy does not compromise patient care.

## Data Availability

The datasets generated and analyzed in the current study are not publicly available due to data protection regulations. Access to data is limited to the researchers who have obtained permission for data processing. Further inquiries can be made to the corresponding author.
